# Novel anesthetic agent remimazolam as an alternative for the asleep-awake-asleep technique of awake craniotomy

**DOI:** 10.1186/s40981-020-00398-5

**Published:** 2020-11-17

**Authors:** Takehito Sato, Yumi Kato, Mayumi Yamamoto, Kimitoshi Nishiwaki

**Affiliations:** Department of Anesthesiology, Nagoya University hospital, 65 Tsurumai-cho, Showa-ku, Nagoya city, Aichi 466-8550 Japan

To the Editor,

Remimazolam is a novel ultrashort-acting benzodiazepine with the advantage of a faster onset and recovery than pre-existing benzodiazepines, such as midazolam [1]. Awake craniotomy (AC) is performed in patients with brain tumors that present in regions linked to language processing, with the aim of minimizing damage to language functioning [2]. There have been reports on administering dexmedetomidine [2], propofol [3, 4], and remifentanil, for the induction of anesthesia in AC; however, there are no reports on administering remimazolam. We describe anesthesia using remimazolam in the asleep-awake-asleep technique of AC.

A 37-year-old man (height 175 cm, weight 58 kg) with a diagnosis of a brain tumor in the right cerebrum, without other medical histories, was scheduled for AC. Anesthesia management comprised a combination of general anesthesia and scalp blocks [4]. General anesthesia was induced with remimazolam (12 mg kg^−1^ h^−1^), remifentanil (0.1 μg kg^−1^ min^−1^), and fentanyl (75 μg). The patient lost consciousness 93 s after starting remimazolam infusion, with a cumulative dose of 20.3 mg. Bispectral Index (BIS) values were approximately 60. Rocuronium (20 mg) was administered, and I-gel® size 5 was inserted. Anesthesia was maintained with remimazolam (1 mg kg^−1^ h^−1^) and remifentanil (0.12–0.15 μg kg^−1^ min^−1^), and the BIS value remained approximately 50 to 60.

Administration of remimazolam and remifentanil was discontinued at the request of the neurosurgeon to awaken the patient. After 26 min, the patient was fully awake, and the I-gel® was removed. He awoke clearly and was cooperative; therefore, language mapping was performed safely. There was no incidence of adverse events such as restlessness, seizure, and pain during the awakening period. Approximately 100 min after arousal, the awake phase was completed; anesthesia was reinduced with remimazolam (12 mg kg^−1^ h^−1^) and remifentanil (0.1–0.12 μg kg^−1^ min^−1^).

The operation was successfully completed, and the I-gel® was removed 26 min after discontinuation of remimazolam. He experienced no delay in arousal or respiratory depression. The total dose of remimazolam was 327 mg. The operating time was 300 min, and the anesthesia time was 463 min.

To date, propofol has primarily been used for anesthesia management of the asleep-awake-asleep technique of AC. However, the use of propofol has disadvantages, such as a risk of developing propofol infusion syndrome [5], vascular pain when infusing, and the absence of antagonist agents. Dexmedetomidine, a highly selective α2 agonist, has been widely used due to its sedative and analgesic effect and ability to preserve spontaneous breathing [2]. However, disadvantages of dexmedetomidine include delayed arousal, cardiovascular effects such as bradycardia and hypotension, and, as with propofol, the absence of an antagonist [4].

In this case, the patient fully awoke and was able to perform intraoperative language tasks during the AC. We suggest remimazolam to be an alternative option for general anesthesia for AC; it is an ultrashort-acting agent, and if delayed arousal occurred, flumazenil can be administered as an antagonist [1, 6].

Remimazolam could be safely and successfully used for anesthetic management of the asleep-awake-asleep technique of AC.

## Data Availability

Not applicable
